# Hemorrhagic Complications in Patients with Atrial Fibrillation Treated with Novel Oral Anticoagulants: Results from the CRAFT Study

**DOI:** 10.3390/jcm15010229

**Published:** 2025-12-27

**Authors:** Marek Styczkiewicz, Mateusz Wawrzeńczyk, Adam Sukiennik, Bartosz Krzowski, Cezary Maciejewski, Piotr Lodziński, Leszek Kraj, Grzegorz Opolski, Marcin Grabowski, Paweł Balsam, Michał Peller

**Affiliations:** 1 Department of Cardiology, The Pope John Paul II Province Hospital of Zamość, 22-400 Zamość, Poland; styczkiewicz@interia.pl; 2 1st Department of Cardiology, Medical University of Warsaw, 02-091 Warsaw, Poland; 3 Department of Oncology, Medical University of Warsaw, 02-091 Warsaw, Poland

**Keywords:** atrial fibrillation, bleeding risk score, anticoagulant therapy, DOACs

## Abstract

**Background/Objectives**: Hemorrhagic complications are among the most common adverse events of anticoagulant therapy in patients with atrial fibrillation (AF). Non-vitamin K antagonist oral anticoagulants (DOACs) are known to be more effective than vitamin K antagonists (VKAs) in preventing thromboembolism. The aim was to identify clinical factors associated with hemorrhagic events in AF patients treated with DOACs and to develop a simple, clinically applicable bleeding risk score. **Methods**: Data were derived from the multicenter CRAFT trial (NCT02987062). We conducted a retrospective analysis of hospital records of 1435 AF patients (median age: 67 years; 44.8% female) treated with dabigatran or rivaroxaban. The main study endpoints were the occurrence of a bleeding episode, thromboembolic episode, or all-cause death during a mean four-year follow-up (1531 [1062–2140] days). **Results**: The rates of bleeding episodes, thromboembolic episodes, and all-cause death were 17.4%, 13.5%, and 23.9%, respectively. Nine factors were identified as predictors of bleeding complications: male sex, history of major bleeding, history of cancer, COPD, CRT, rivaroxaban therapy, statin therapy, age, and absence of heart failure. Based on these, the CRAFT bleeding score was developed to predict the risk of hemorrhagic events in individual patients. **Conclusions**: The CRAFT bleeding score may be implemented in AF patients as an additional tool for evaluating DOACs safety prior to initiating anticoagulant therapy, and for guiding closer monitoring of high-risk individuals to minimize bleeding complications.

## 1. Introduction

Anticoagulant therapy is essential for patients with atrial fibrillation (AF), as it prevents thrombotic complications, most importantly ischemic stroke. Except for patients at very low thrombotic risk, it is widely used [1,2,3]. However, it carries well-recognized side effects such as hemorrhagic complications.

In recent years, the management of atrial fibrillation has evolved substantially with the introduction of non-vitamin K antagonist oral anticoagulants (DOACs), including dabigatran, rivaroxaban, apixaban, and edoxaban. DOACs have been shown to provide several advantages over vitamin K antagonists (VKAs), both in preventing thromboembolic episodes and reducing the frequency of bleeding complications [4,5,6]. These agents provide predictable pharmacokinetics, fewer drug–food interactions, and do not require routine INR monitoring, which has significantly improved patient convenience. However, despite their favorable safety profile compared to warfarin, bleeding events remain a relevant clinical concern. Several bleeding risk scores have been proposed, such as HAS-BLED and, more recently, the DOAC score, but these were derived mainly from VKA-treated populations and may not fully capture risk factors specific to DOAC therapy [7,8]. Therefore, developing a DOAC-specific bleeding risk score based on real-world data is important to better individualize treatment and minimize complications.

The aim of this study was to evaluate factors influencing bleeding risk and to develop a reliable clinical score to assess the risk of hemorrhagic events in patients treated with DOACs.

## 2. Materials and Methods

### 2.1. Study Design

Data were obtained from the MultiCenter expeRience in AFib patients Treated with oral anticoagulation (CRAFT, NCT02987062) study. The CRAFT registry was designed as a retrospective, multicenter, observational cohort study involving two Polish hospitals (one academic tertiary-care center and one regional hospital).

The study included adult patients with AF hospitalized between 2011 and 2016 in one of two hospitals: one academic and one regional. Details have been reported elsewhere [9]. Patients diagnosed with valvular AF or not treated with OACs were not included in the study. Patients treated with apixaban were excluded from the study due to small group size, meanwhile edoxaban was not available in Poland during that period, therefore patients included in the study were treated with dabigatran or rivaroxaban. Baseline characteristics regarding demographics, medical history, type of AF, diagnostic tests results and pharmacotherapy were collected.

The primary endpoints were the occurrence of a bleeding episode, thromboembolic episode, or all-cause death during the mean follow-up of four years (1531 [1062–2140] days). Bleeding events were identified using diagnostic codes for gastrointestinal, intracranial, and other bleeding-related conditions. Ischemic events included ischemic stroke, transient ischemic attack, and peripheral thromboembolism. Due to the retrospective nature of the study, some parameters were not available for all patients.

### 2.2. Statistical Analysis

All statistical analyses were conducted using SAS 9.4 (SAS Institute, Cary, NC, USA). Continuous variables were tested for normality using the Shapiro–Wilk test and presented as mean ± standard deviation or median with interquartile range, depending on distribution. Categorical variables were reported as counts and percentages.

Comparisons between groups were performed using the chi-square or Fisher’s exact test for categorical variables, and Student’s t-test or Mann–Whitney U test for continuous variables, depending on distribution. Time-to-event outcomes were analyzed using Kaplan–Meier estimates and compared between groups using the log-rank test.

To identify independent predictors of hemorrhagic events, a multivariate Cox proportional hazards regression model was constructed. Variables with a *p*-value < 0.10 in univariate analysis were entered into the multivariate model. Hazard ratios (HRs) with 95% confidence intervals (CIs) were calculated. A two-sided *p*-value < 0.05 was considered statistically significant.

Based on the multivariate model, a clinical scoring system was developed to stratify patients by bleeding risk. Predictors were assigned weighted point values reflecting their relative contributions. Discriminatory performance was assessed using receiver operating characteristic (ROC) curve analysis. Patients were categorized into low-, medium-, and high-risk groups.

All statistical tests were two-tailed and performed at a significance level of α = 0.05

## 3. Results

A total of 1435 patients were included (median age: 67 years; 44.8% female). Over a mean follow-up of 4.2 years, bleeding events occurred in 17.4%, thromboembolic events in 13.5%, and death in 23.9%.

Several factors significantly influenced the incidence of bleeding complications. Congestive heart failure and female sex reduced the risk of bleeding, while seven factors increased risk: history of major bleeding, history of cancer, COPD, CRT, rivaroxaban therapy, statin therapy, and age. Baseline characteristics and the number of patients analyzed for each parameter are shown in Table 1. Results and statistical data are presented in Table 2.

Among these, two factors had the most significant effect: a history of major bleeding, which increased the risk of hemorrhage more than threefold, and CRT, which increased the risk nearly fivefold. Conversely, congestive heart failure reduced the incidence of bleeding events by nearly half.

Based on these findings, the CRAFT bleeding score was developed to determine the risk of hemorrhagic complications. The scoring system is presented in Table 3.

**Figure 1:**
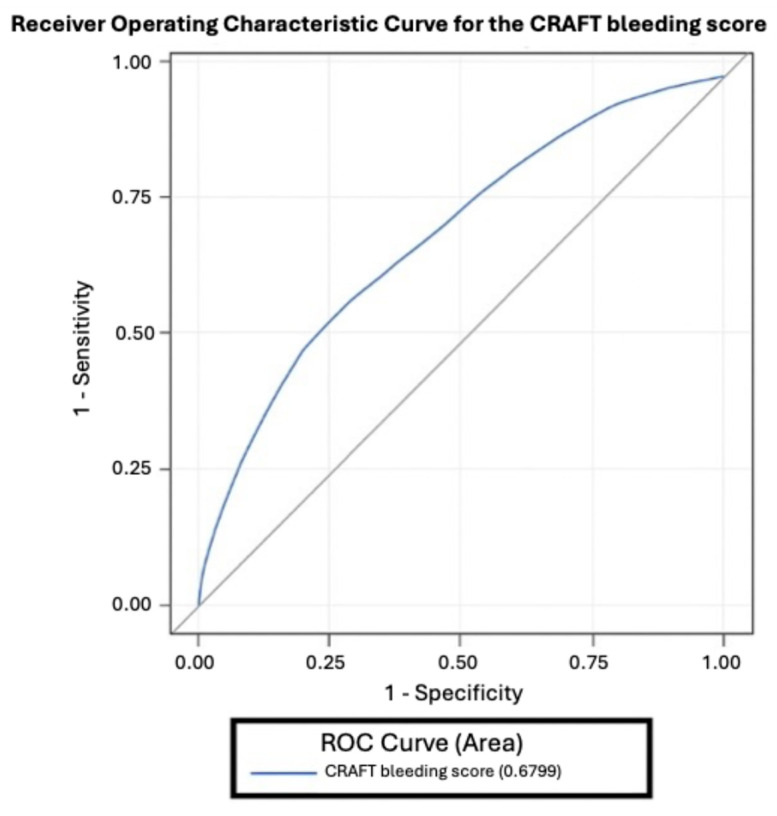
Receiver operating characteristic curve for the CRAFT bleeding score; ROC—receiver operating characteristic.

The ROC curve (Figure 1) demonstrates good discriminatory ability of the score in predicting hemorrhagic episodes. Patients were stratified into three groups: Low risk (≤10 points), Medium risk (11–14 points), and High risk (≥15 points).

Table 4 shows the distribution of patients by risk group. The risk of bleeding was less than 7% in group 1, but reached 38% in group 3, underscoring the clinical utility of the score.

## 4. Discussion

Based on real-life registry data, we developed a practical score for predicting bleeding risk in patients receiving DOAC therapy for AF. Importantly, the CRAFT bleeding score relies solely on clinical history, without requiring additional laboratory tests, making its application simple and widely accessible.

Most parameters identified in this study were consistent with expectations. History of hemorrhage, older age, and cancer were clearly associated with increased bleeding risk [10,11]. CRT also emerged as a strong risk factor; however, heart failure appeared to reduce bleeding risk, which is somewhat counterintuitive. A plausible explanation is that patients with HF—particularly those with implanted cardiac devices—tend to receive more structured follow-up and closer home-based surveillance, which may limit exposure to uncontrolled hypertension or inappropriate medication use. Matteucci Et Al. demonstrated that telemonitoring in HF patients allows early identification of clinical deterioration and timely optimization of therapy [12]. In their cohort of 312 individuals, remote monitoring during the COVID-19 lockdown resulted in fewer emergency admissions for arrhythmic and HF events compared with the pre-pandemic period, despite an increased number of HF-related alerts. This supports the notion that systematic surveillance can stabilise clinical status and reduce the likelihood of acute changes in pharmacotherapy that might predispose to bleeding. Such close monitoring also facilitates regular reassessment of anticoagulant indication and dosing, as well as prompt detection of laboratory abnormalities that contribute to bleeding risk.

Findings from Ikebe Et Al. further highlight the importance of HF severity in shaping prognosis [13]. In a large PCI cohort, HF accompanied by elevated BNP levels was independently associated with higher rates of major bleeding, subsequent major adverse cardiac events, and all-cause mortality. Moreover, early post-procedural bleeding markedly amplified this risk. These results indicate that any potential attenuation of bleeding risk in HF patients is more likely a consequence of intensified clinical oversight than a reflection of HF biology. Only in settings where follow-up is rigorous and anticoagulation is closely supervised might HF appear to confer a relative reduction in bleeding events.

Taken together, available evidence suggests that HF itself is a marker of heightened vulnerability and increased bleeding risk. The apparently protective effect observed in our DOAC-treated cohort is therefore best interpreted as a “surveillance effect,” driven by more vigilant monitoring, cautious dose adjustment, and overall tighter long-term management in this patient population. 

The development of a DOAC-specific prediction tool is crucial, as most existing scores were derived from VKA-treated cohorts [14] and may not accurately capture DOAC-related bleeding risk [7]. Compared with other scores, ours appears more effective, being derived exclusively from DOAC-treated patients.

The widely used HAS-BLED score was developed largely from warfarin-treated populations [7]. It does not consider factors such as CRT, rivaroxaban use, or sex. Moreover, one HAS-BLED component is labile INR, which is irrelevant for DOAC therapy. While HAS-BLED performs well in identifying bleeding risk among multimorbid patients with liver dysfunction or hypertension, it may underestimate risk in otherwise healthy patients undergoing rivaroxaban therapy or CRT, potentially leading to unexpected hemorrhagic events.

The CRAFT bleeding score incorporates several significant factors absent from the recently proposed DOAC score [8], notably CRT, which increased bleeding risk nearly fivefold in our study. For example, an elderly patient treated with rivaroxaban, undergoing CRT, and suffering from COPD would be classified as very high risk by the CRAFT score, while the DOAC score might not reach the same conclusion. However, it must be noted that the DOAC score was developed in more diverse cohorts treated with multiple DOACs (dabigatran, rivaroxaban, apixaban, edoxaban), which may make it more broadly applicable outside our population.

The CRAFT bleeding score stratifies patients into low-, medium-, and high-risk groups, with good correlation between score and bleeding incidence. This has several clinical benefits: it allows identification of high-risk patients, enabling treatment modification (e.g., dose reduction, switching to another DOAC) to reduce bleeding risk. It may also guide closer monitoring and preventive care, thereby reducing bleeding-related mortality. In particular, patients at high risk of bleeding may benefit from switching from rivaroxaban to another DOAC. Apixaban, for example, is associated with a lower bleeding risk compared to rivaroxaban [15,16], and may be a better option for such patients.

The CRAFT bleeding score has several potential clinical applications. It can serve as a practical tool for physicians to identify AF patients at increased risk of hemorrhagic events before initiating DOAC therapy, supporting personalized decision-making. In high-risk patients, clinicians may consider avoiding rivaroxaban, therefore selection of a DOAC with a lower bleeding profile, such as apixaban. Furthermore, the CRAFT score could be integrated into electronic health record systems and clinical decision-support software, enabling automated, real-time risk assessment during routine clinical visits. Ultimately, its implementation may improve patient safety, adherence to therapy, and long-term treatment outcomes in anticoagulated AF populations.

It is worth noting that non-anticoagulation strategies may also be considered for stroke prevention especially in growing population of patients diagnosed with wearable technologies and recordings from cardiovascular implantable electronic devices (CIEDs) [17]. Those strategies may include drugs such as folic acid, rosuvastatin, and candesartan in patients with low cardiovascular risk. Moreover treatment with GLP-1) receptor agonists may reduce risk of stroke in diabetic patients [18,19], while in CAD patients colchicine has stroke-preventative potential [20].

A major limitation of the CRAFT score is the absence of external validation. Future studies must evaluate the score prospectively in larger, multicenter cohorts that include patients treated with apixaban and edoxaban. This will allow proper assessment of the model’s discrimination and calibration and will position CRAFT directly against established tools such as HAS-BLED and the DOAC score.

Integrating this score into clinical decision-support systems has a potential to facilitate automatic risk stratification at the point of care. Moreover, combining bleeding and thromboembolic risk models could enable more personalized anticoagulation strategies.

## 5. Conclusions

Among the factors significantly increasing bleeding risk during DOAC therapy, the strongest predictors were history of bleeding and CRT. In patients with these risk factors, heightened vigilance and close monitoring should be implemented.

Based on data from the CRAFT registry, we developed the CRAFT bleeding score, which stratifies patients into three risk categories and may serve as a clinically useful tool to identify patients at increased risk of hemorrhagic events. It may guide treatment modification and closer follow-up for individuals at high risk, therefore improving patient safety and treatment outcomes. Future validation in prospective studies will determine its role in everyday clinical practice.

## Figures and Tables

**Table 1:** Baseline characteristic and follow-up outcomes.

Variable	Value	Number of Patients in Which the Parameter Was Assessed
**Demographics**		
**Age [years]**	67 [IQR 62–71]	1435
**Female**	643 (44.8%)	1435
**Comorbidities**		
**CHF**	532 (37.1%).	1433
**HT**	1022 (71.3%).	1434
**CAD**	611 (42.6%).	1435
**Vascular disease**	615 (42.9%).	1435
**DM2**	370 (25.9%).	1430
**History of stroke/TIA**	216 (15.1%).	1431
**COPD**	145 (10.1%).	1433
**History of hemorrhagic event**	131 (9.1%)	1435
**History of neoplastic disease**	84 (8.5%).	992
**PM**	266 (26.9%).	990
**ICD**	55 (5.5%).	992
**CRT**	14 (1.4%).	992
**AF type**		
**Persistent AF**	192 (14.1%).	1361
**Permanent AF**	356 (26.2%).	1361
**Medications**		
**Rivaroxaban**	968 (67.5%)	1435
**Dabigatran**	467 (32.5%)	1435
**Antiplatelet drugs**	137 (9.6%).	1435
**ASA**	110 (7.7%)	1435
**Clopidogrel**	54 (5.4%)	992
**Antiarrhythmic drugs**	249 (17.4%)	1433
**Beta-blockers**	784 (79.0%)	992
**ACEI/ARB**	696 (70.1%)	992
**Statins**	609 (61.4%)	992
**Calcium channel blockers**	233 (23.5%)	992
**Others**		
**SBP >160 mmHg**	38 (3.8%).	992
**Regular alcohol consumption**	21 (1.5%).	1431
**Follow-up outcomes—MAEs**		
**Hemorrhagic event**	250 (17.4%)	1435
**Thromboembolic event**	194 (13.5%)	1435
**Death**	343 (23.9%)	1435

ACEI—angiotensin-converting-enzyme inhibitors; AF—atrial fibrillation; ARB—angiotensin receptor blockers; ASA—acetylsalicylic acid; CAD—coronary artery disease; COPD—chronic obstructive pulmonary disease; CHF—congestive heart failure; CRT—cardiac resynchronization therapy; DM2—diabetes mellitus type 2; HT—hypertension; ICD—implantable cardioverter-defibrillator; MAEs—major adverse events; PM—pacemaker; SBP—systolic blood pressure; TIA—transient ischemic attack.

**Table 2:** Factors influencing the risk of bleeding complications.

Parameter	Hazard Ratio	Hazard Ratio (95% CI)	*p*-Value
**Female sex**	0.665	0.472–0.937	0.0198
**History of hemorrhagic event**	3.136	1.671–5.885	0.0004
**History of cancer**	1.799	1.113–2.906	0.0164
**Congestive heart failure**	0.528	0.344–0.812	0.0036
**COPD**	2.341	1.434–3.819	0.0007
**Cardiac resynchronization therapy**	4.802	1.662–13.875	0.0037
**Rivaroxaban**	1.524	1.041–2.232	0.0304
**Statin**	1.442	1.015–2.049	0.0412
**Age**	1.042	1.027–1.058	<0.0001

CI—confidence interval; COPD—chronic obstructive pulmonary disease.

**Table 3:** A CRAFT bleeding score predicting the bleeding complications based on the CRAFT registry.

Parameter	Points
**Cardiac resynchronization therapy**	5
**History of hemorrhagic event**	3
**Male sex**	2
**History of cancer**	2
**Absence of heart failure**	2
**COPD**	2
**Rivaroxaban**	2
**Statin**	1
**Age**	Number of years/10 years

COPD—chronic obstructive pulmonary disease.

**Table 4:** Stratification of the patients according to the bleeding risk based on the CRAFT bleeding score results.

Groups		Absence of Event	Presence of Event	Total
**Low-risk** **≤10 points**	Number of patients	257	19	276
	Percent of total patients	25.91%	1.92%	27.82%
	Percent of group	93.12%	6.88%	
**Medium-risk** **11–14 points**	Number of patients	491	100	591
	Percent of total patients	49.50%	10.08%	59.58%
	Percent of group	83.08%	16.92%	
**High-risk** **≥15 points**	Number of patients	77	48	125
	Percent of total patients	7.76%	4.84%	12.6%
	Percent of group	61.60%	38.40%	
**Total**	Number of patients	825	167	992
	Percent of total patients	83.17%	16.83%	

## Data Availability

The original contributions presented in this study are included in the article. Further inquiries can be directed to the corresponding author.
